# A Postbiotic‐Inducing *Ganoderma applanatum* Polysaccharide Repairs the Intestinal Barrier via a *Phocaeicola vulgatus ‐* Tridecylic Acid Axis

**DOI:** 10.1002/advs.78105

**Published:** 2026-10-06

**Authors:** Chuan Zhang, Miaoyu Li, Tiantian Li, Xiaojie Zang, Haiyang Shi, Yaru Liu, Chengcheng Zhang, Jianxin Zhao, Qixiao Zhai, Wei Chen, Fengwei Tian

**Affiliations:** ^1^ State Key Laboratory of Food Science and Resources Jiangnan University Wuxi Jiangsu People's Republic of China; ^2^ School of Food Science and Technology Jiangnan University Wuxi Jiangsu People's Republic of China; ^3^ Center For General Practice Medicine Department of General Practice Medicine Zhejiang Provincial People's Hospital Affiliated People's Hospital Hangzhou Medical College Hangzhou Zhejiang People's Republic of China; ^4^ National Engineering Research Center for Functional Food Jiangnan University Wuxi Jiangsu People's Republic of China

**Keywords:** barrier function, ganoderma applanatum, gut microbiota, intestinal mucosal barrier, *phocaeicola vulgatus*, polysaccharide

## Abstract

Intestinal barrier dysfunction is a pathological hallmark of ulcerative colitis (UC), yet therapeutic strategies that precisely restore epithelial integrity remain limited. Here, we identified four distinct polysaccharide fractions from *Ganoderma applanatum* (GAPs) and pinpointed GAP2 as the principal bioactive component that significantly ameliorates DSS‐induced colitis and intestinal barrier injury. Furthermore, experiments using germ‐free mice and fecal microbiota transplantation show that the barrier‐repairing efficacy of GAP2 depends on the gut microbiota and their metabolic outputs. This indicates that GAP2 acts as a microbiota‐directed modulator, exerting health benefits by enriching *Phocaeicola vulgatus* and tridecylic acid. *P. vulgatus* degrades GAP2 via GH16, facilitating fatty‐acid metabolism to generate tridecanoic acid (absent in the mutant), which may activate the Rho/CDC42 pathway in intestinal epithelial cells to promote tight‐junction assembly, thereby restoring barrier integrity. Notably, a meta‐analysis of UC patient cohorts revealed that both the abundance of *P. vulgatus* and its capacity for tridecylic acid biosynthesis were significantly decreased. Our findings establish GAP2 as a potent postbiotic inducer that repairs the intestinal barrier via a potential *P. vulgatus*‐tridecylic acid‐Rho/CDC42 axis. This work highlights the therapeutic potential of targeting this newly identified microbe‐metabolite‐signaling axis for the treatment of UC and related mucosal inflammatory disorders.

## Graphical abstract

This study identifies GAP2, a bioactive polysaccharide fraction derived from *Ganoderma applanatum*, as a postbiotic inducer that repairs intestinal barrier damage in a gut microbiota‐dependent manner. GAP2 enriches *Phocaeicola vulgatus*, which degrades GAP2 and promotes tridecylic acid production, triggering Rho/CDC42 signaling and tight‐junction assembly to improve ulcerative colitis pathology. GAP2 and *Phocaeicola vulgatus* could be promising therapeutic candidates for ulcerative colitis.

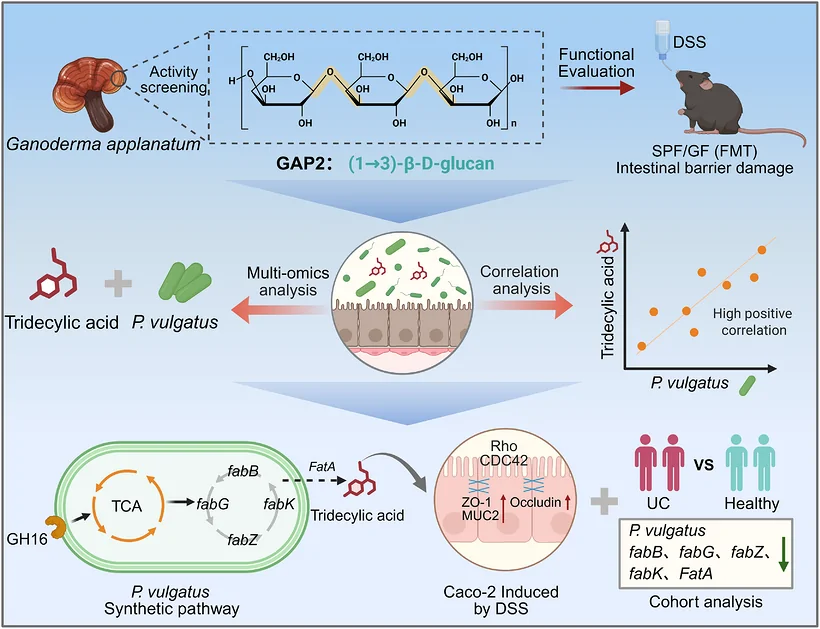

## 1 Introduction

The intestinal barrier serves as a critical defense system for maintaining host homeostasis by segregating luminal contents from the mucosal immune environment. This separation facilitates selective nutrient absorption while restricting the translocation of bacteria, antigens, and other potentially harmful factors across the epithelium into the lamina propria [1, 2, 3]. The integrity of this barrier depends on intercellular junctional complexes composed of proteins including zonula occludens‐1 (ZO‐1), occludins, and claudins [4]. Disruption of tight junction structure or function leads to increased intestinal permeability, a condition that promotes mucosal inflammatory responses. Such impairment significantly contributes to the initiation and progression of inflammatory bowel disease (IBD), including both Crohn's disease (CD) and ulcerative colitis (UC) [5, 6]. Accordingly, strategies aimed at restoring intestinal barrier integrity and regulating tight junction homeostasis may provide valuable avenues for identifying new therapeutic targets and developing more effective interventions for UC.

Gut microbiota have garnered widespread attention due to their involvement in host metabolism and multifaceted physiological regulation [7, 8]. In the context of intestinal inflammation, dysbiosis is characterized by the overgrowth and colonization of endotoxin‐producing pathogenic bacteria, alongside a reduction in various protective commensals such as *Bacteroides*, *Alistipes*, *Parabacteroides*, *Ruminococcus*, *Roseburia*, *Lactobacillus*, *Bifidobacterium*, and *Akkermansia* [5, 9, 10, 11]. The microbiota exerts systemic effects on the host primarily through its metabolites, which interact with intestinal epithelial cells to regulate epithelial renewal and immune homeostasis, thereby preserving barrier integrity [12, 13]. In UC, for instance, decreased abundance of Firmicutes and *Lachnospiraceae* is associated with reduced production of short‐chain fatty acids (SCFAs), compromising epithelial energy supply and tight junction homeostasis. This leads to increased permeability and amplified inflammatory responses, ultimately exacerbating barrier injury [13, 14]. Recent studies suggest that ginseng polysaccharides can ameliorate intestinal barrier dysfunction and alleviate inflammation by remodeling microbiota composition and metabolic functions, particularly by promoting SCFAs production [15]. For example, polysaccharides from *Gracilaria lemaneiformis* restore beneficial bacteria such as *Lactobacillus* and enhance SCFAs generation, thereby mitigating DSS‐induced colitis [16]. *Cyclocarya paliurus* polysaccharides restored gut microbial homeostasis by enriching beneficial *Bacteroidetes* and enhancing SCFAs production [17]. Fucoidan increases the abundance of *Muribaculum* sp002492595, reinforces barrier integrity, reduces bacterial translocation, and promotes intestinal homeostasis [14]. Nevertheless, the critical functional nodes and regulatory networks linking natural polysaccharides, gut microbiota metabolism, and barrier function remain to be systematically elucidated. This underscores that targeting microbial metabolic pathways to facilitate barrier repair may represent a pivotal strategy for intervening in UC and preserving intestinal health.

*Ganoderma applanatum* (*G. applanatum*) is a globally recognized medicinal and functional food, long utilized in traditional medicine and health products [18, 19]. Enriched with bioactive constituents such as polysaccharides, flavonoids, and triterpenoids, it exhibits immunomodulatory, anti‐inflammatory, antioxidant, and metabolic regulatory properties [20, 21]. *Ganoderma applanatum* polysaccharides (GAPs) can serve as prebiotics, enhancing intestinal metabolism and selectively enriching commensal microbiota to improve gut health [22]. Our previous studies have demonstrated that GAPs mitigate pathogen infection and UC by modulating microbial structure and metabolite profiles [23]. However, it remains unclear which subfractions of biologically active GAPs are more effective for barrier protection; whether the key mechanisms primarily involve the epithelial tight junction complex, mucosal barrier repair, inflammatory signaling pathways, or the microbiota–metabolite axis; and whether a clear “structure–function” relationship exists. Therefore, identifying the bioactive components of GAPs and the key gut microbiota and metabolites they regulate is of substantial significance.

This research aimed to elucidate how specific constituents of GAPs influenced UC pathology, particularly the restoration of epithelial barrier integrity. We demonstrated that the beneficial effects of GAPs on intestinal barrier dysfunction depend on postbiotics derived from the gut microbiota metabolism of GAPs. Furthermore, we identified that the active component of GAP2 reshapes *P. vulgatus* and its fatty acid metabolism pathways to generate tridecylic acid, which was identified as a key mediator conferring the benefits of GAP2, primarily via activation of the CDC42/Rho signaling pathway. Importantly, correlational evidence from clinical datasets supports the relevance of this microbe‐associated metabolic potential to active UC. Collectively, these findings provide mechanistic support for targeting microbiota‐dependent signaling circuits as a strategy to alleviate intestinal barrier dysfunction in UC and related inflammatory mucosal disorders.

## 2 Results

### 2.1 *Ganoderma applanatum* Polysaccharides Exhibited a Component‐Specific Protective Effect on DSS‐Induced Intestinal Barrier Injury in Mice

Previous studies have shown that water‐extracted polysaccharides from *G. applanatum* exert beneficial effects on intestinal inflammation and barrier dysfunction; however, their specific active fractions remain unclear [24, 25]. To identify these active components, we performed isolation and purification of water‐extracted polysaccharides from *G. applanatum* (Figure 1A). The polysaccharides were purified using anion‐exchange and gel filtration chromatography, yielding four major fractions: the water‐eluted fraction, the 0.2 mol/L NaCl‐eluted fraction, the 0.5 mol/L NaCl‐eluted fraction, and the 1.0 mol/L NaCl‐eluted fraction. Based on the elution order, these fractions were designated GAP1, GAP2, GAP3, and GAP4, respectively (Figure 1B). All fractions, including the crude polysaccharide control, significantly alleviated multiple clinical indices in DSS‐induced colitis mice, such as body weight loss, colon shortening, and disease activity index (DAI) (Figure 1C–E). In the evaluation of intestinal barrier‐related enzyme activities and inflammatory markers, GAP1 and GAP2 significantly inhibited the activities of inducible nitric oxide synthase (iNOS), myeloperoxidase (MPO), and cyclooxygenase‐2 (COX‐2) (Figure S1A–C), markedly reduced the concentrations of pro‐inflammatory cytokines, including IL‐1β, IL‐6, IL‐17, and TNF‐α, while increasing the secretion of the anti‐inflammatory cytokine IL‐10 (Figure S1D–H), suggesting their dual roles in anti‐inflammation and immunoregulation. Regarding intestinal barrier‐related proteins, only GAP1 and GAP2 significantly modulated barrier function‐related indicators, indicating their direct effects on barrier repair (Figure 1F). Histological evidence from H&E and PAS staining further supported that GAP1 and GAP2 significantly alleviated mucosal injury, inflammatory cell infiltration, and glandular structural damage, while improving the integrity of mucopolysaccharide‐ and glycogen‐related structures (Figure 1G). Taken together, this study is the first to demonstrate that GAP1 and GAP2 are the active constituents of GAPs that ameliorate intestinal inflammation and barrier dysfunction.

**FIGURE 1:**
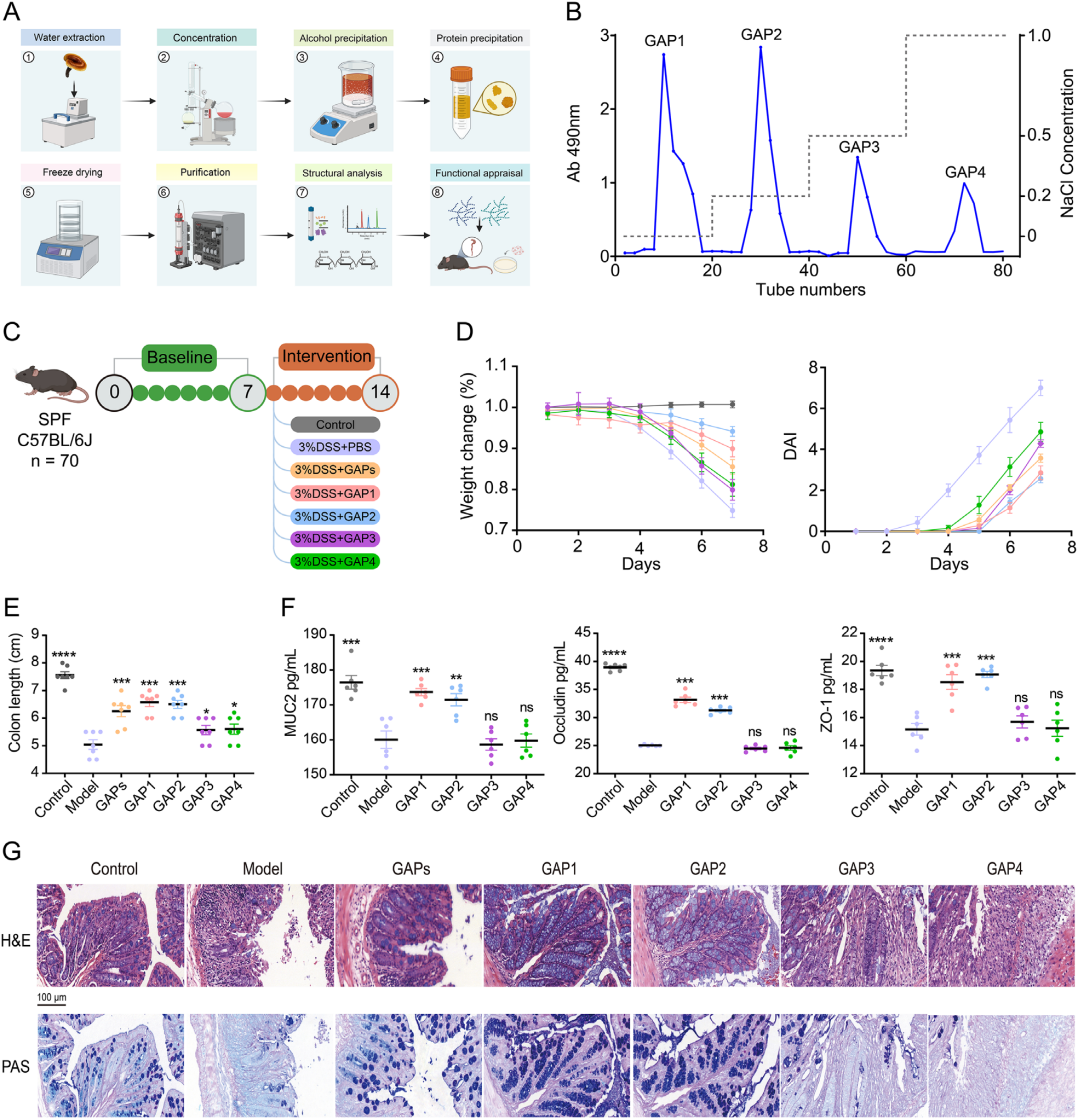
Isolation, purification, and intestinal barrier activity regulation of GAPs. (A) Flowchart illustrating the extraction and efficacy evaluation of GAPs. (B) The elution curve of HiTrap DEAE Fast Flow chromatography of crude GAPs. (C) Schematic diagram of animal experiments designed to evaluate the efficacy of GAPs. (D,E) Changes in body weight, colon length and disease severity score of DSS‐treated mice with polysaccharide intervention (*n* = 7 per group). (F) Changes in the content of tight junction proteins in the colon tissue from DSS‐treated mice with polysaccharide intervention (*n* = 6 per group). (G) H&E and PAS staining of the colon of DSS‐treated mice with polysaccharide intervention. One‐way analysis of variance (ANOVA) followed by Tukey's test was used for gene expression analysis. “ns” indicates *p* > 0.05; ^*^ indicates *p* < 0.05; ^**^ indicates *p* < 0.01; ^***^ indicates *p* < 0.001 and ^****^ indicates *p* < 0.0001.

### 2.2 The Effective Bioactive Components GAP1 and GAP2 Both Contain a Glucan Backbone

To elucidate the glycosidic linkage types and structural characteristics of GAP1 and GAP2, comprehensive analyses were performed using molecular weight determination, monosaccharide composition, fourier‐transform infrared spectroscopy (FT‐IR), and nuclear magnetic resonance (NMR) spectroscopy [26]. Monosaccharide composition analysis revealed that GAP1 primarily consisted of mannose, glucose, and galactose, with molar proportions of 5.42%, 82.69%, and 11.88%, respectively, whereas GAP2 was mainly composed of glucose (Figure S2). Molecular weight and purity were analyzed by high‐performance gel‐permeation chromatography (HPGPC). Both GAP1 and GAP2 exhibited a single symmetric peak, indicating that they were relatively homogeneous polysaccharides with average molecular weights of 1.9033 × 10^4^ Da and 2.0537 × 10^4^ Da, respectively (Figure S3A,B). FT‐IR spectra displayed obvious C–OH stretching vibration peaks in the region of 1200‐1000 cm^−^^1^, further confirming that GAP1 and GAP2 were polysaccharide compounds (Figure S3C,D). NMR analysis of GAP1 revealed that in the ^1^H NMR spectrum, anomeric hydrogen signals appeared at δ 4.54–5.41 ppm, with discrete anomeric signals at 4.54, 5.01, 5.08, 5.24, 5.39, and 5.41 ppm (Figure 2A). In the ^1^ ^3^C NMR spectrum, anomeric carbon resonances were observed at δ 90–103 ppm, with characteristic signals at 91.92, 99.58, 97.88, and 102.72 ppm (Figure 2B). The main linkages and sequences between different residues in GAP1 were further investigated and identified by heteronuclear single quantum coherence (HSQC) and heteronuclear multiple bond correlation (HMBC) (Figure 2C; Figure S3E). Cross‐peaks between H‐1 of residue B and C‐4 of residue A; H‐1 of residue F and C‐4 of residue C/D; C‐1 of residues A and E and H‐4; C‐1 of residue C and H‐4 of residue B; and C‐1 of residues B and F indicated the following connections: →3,6)‐β‐Glcp‐(1→, →3,6)‐β‐Glcp‐(1→, →4,6)‐α‐Glcp‐(1→, →3,6)‐β‐Glcp‐(1→; →2)‐α‐D‐Manp‐(1→, →3,6)‐β‐Glcp‐(1→; →4,6)‐α‐Glcp‐(1→, →3)‐α‐Glcp‐(1→, →4)‐α‐Gal‐(1→; →6)‐α‐D‐Glcp‐(1→, →3,6)‐β‐Glcp‐(1→. NMR analysis of GAP2 showed that in the ^1^H NMR spectrum, the anomeric hydrogen signal was located at δ 4.54 ppm (Figure 2E). In the ^1^ ^3^C NMR spectrum, the anomeric carbon signal was detected at 102.79 ppm (Figure 2F). The main linkages and sequences between residues in GAP2 were further determined by HSQC and HMBC (Figure 2G; Figure S3F). Only a cross‐peak between H‐1 and C‐3 of residue A was detected, indicating that GAP2 is a glucan featuring a →3)‐β‐Glcp‐(1→ backbone.

**FIGURE 2:**
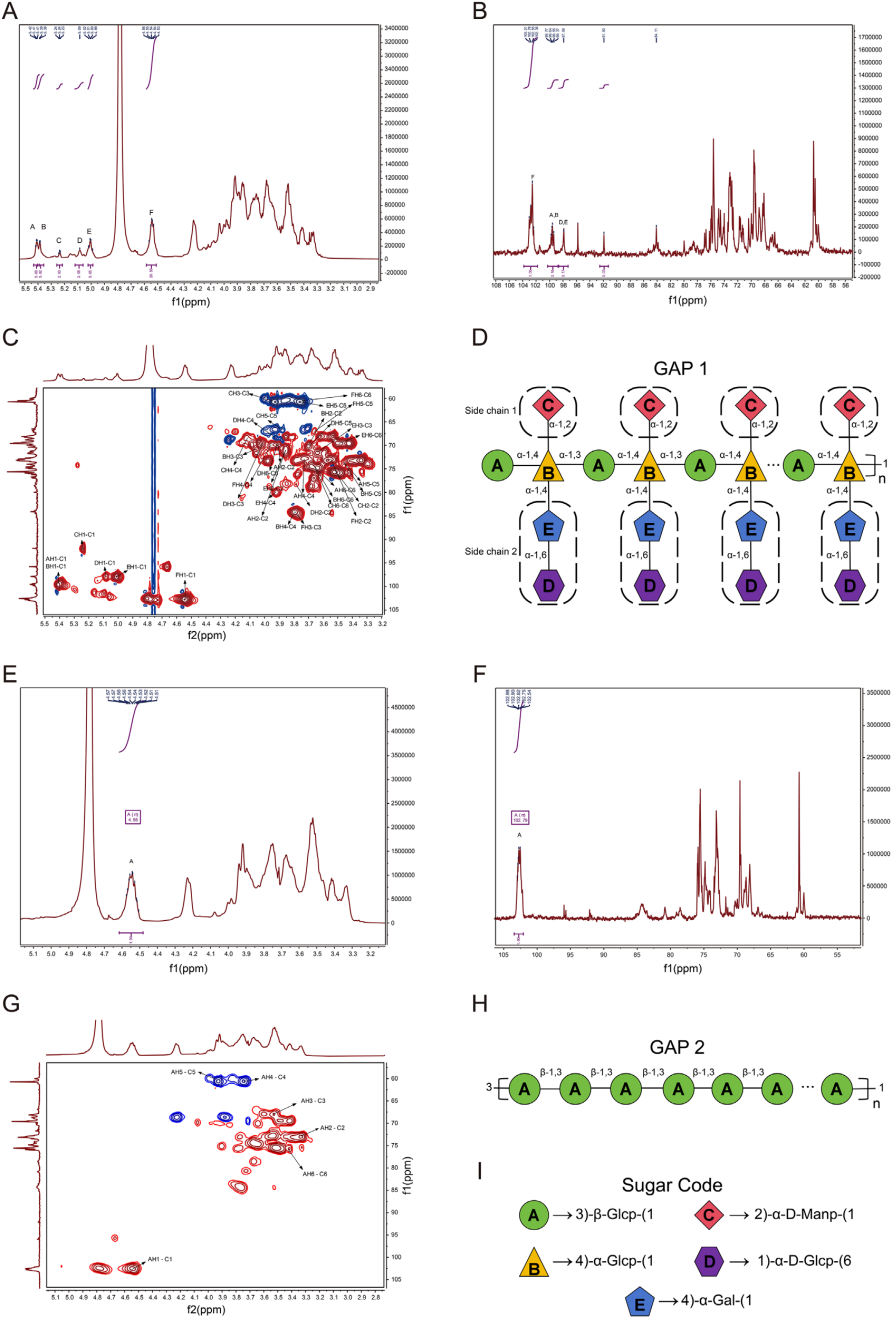
Structural characterization of GAP1 and GAP2. (A,B) One‐dimensional nuclear magnetic resonance spectra of GAP1, ^1^H NMR and ^13^C NMR. (C) Two‐dimensional nuclear magnetic resonance spectra of GAP1, HSQC. (D) The deduced structure of GAP 1 (E,F) One‐dimensional nuclear magnetic resonance spectra of GAP2, ^1^H NMR and ^13^C NMR. (G) Two‐dimensional nuclear magnetic resonance spectra of GAP2, HSQC. (H) The deduced structure of GAP 2. (I) The polysaccharide composed of sugar code.

Taken together, these results demonstrate that the primary structure of GAP1 consiste of a backbone of →3)‐β‐Glcp‐(1→4)‐α‐Glcp‐(1→, with branches of →2)‐α‐D‐Manp‐(1→ attached at O‐6, →4)‐α‐Glcp‐(1→4)‐α‐Gal‐(1→ attached at O‐6, and →6)‐α‐D‐Glcp‐(1→ moieties. In contrast, GAP2 is a glucan with a linear backbone of →3)‐β‐Glcp‐(1→ (Figure 2D,H,I; Tables S1 and S2).

### 2.3 GAP1 And GAP2 Induce Microbe‐Derived Postbiotics to Alleviate Intestinal Barrier Injury

To investigate whether the effects of GAP1 and GAP2 depend on the gut microbiota, we used germ‐free mice that received FMT [27] from mice treated with GAPs, serving as the ulcerative colitis (UC) model (Figure 3A). Compared with the model group, GAP1 and GAP2 failed to significantly mitigate DSS‐induced body weight loss or colon shortening (Figure 3B). Notably, the FMT group significantly reversed colon shortening, with the effect reaching strong statistical significance (*p* < 0.001). Furthermore, analysis of colon pathological sections showed that GAP1 and GAP2 did not alleviate DSS‐induced colitis‐related pathological changes, suggesting that their barrier‐protective effects may depend on the gut microbiota (Figure 3C). Specifically, H&E staining revealed that, compared with the model, GAP1, and GAP2 groups, the FMT‐1 and FMT‐2 groups significantly reduced focal lymphocyte infiltration (LPL); periodic‐acid schiff (PAS) staining showed that the abundance of mucin and mucin‐producing cells (blue) in the FMT groups was significantly increased. GAP1 and GAP2 did not mitigate DSS‐induced inflammatory responses associated with colitis and showed no significant effects on COX‐2, iNOS, or MPO activities, nor on MUC2 levels or the expression of occludin and ZO‐1, suggesting that their barrier‐protective effects may not be independent (Figure S4A,B). In contrast, the FMT‐1 and FMT‐2 groups significantly restored the relevant efficacy, demonstrating that GAP1 and GAP2 can restore the intestinal barrier in the presence of gut microbiota.

**FIGURE 3:**
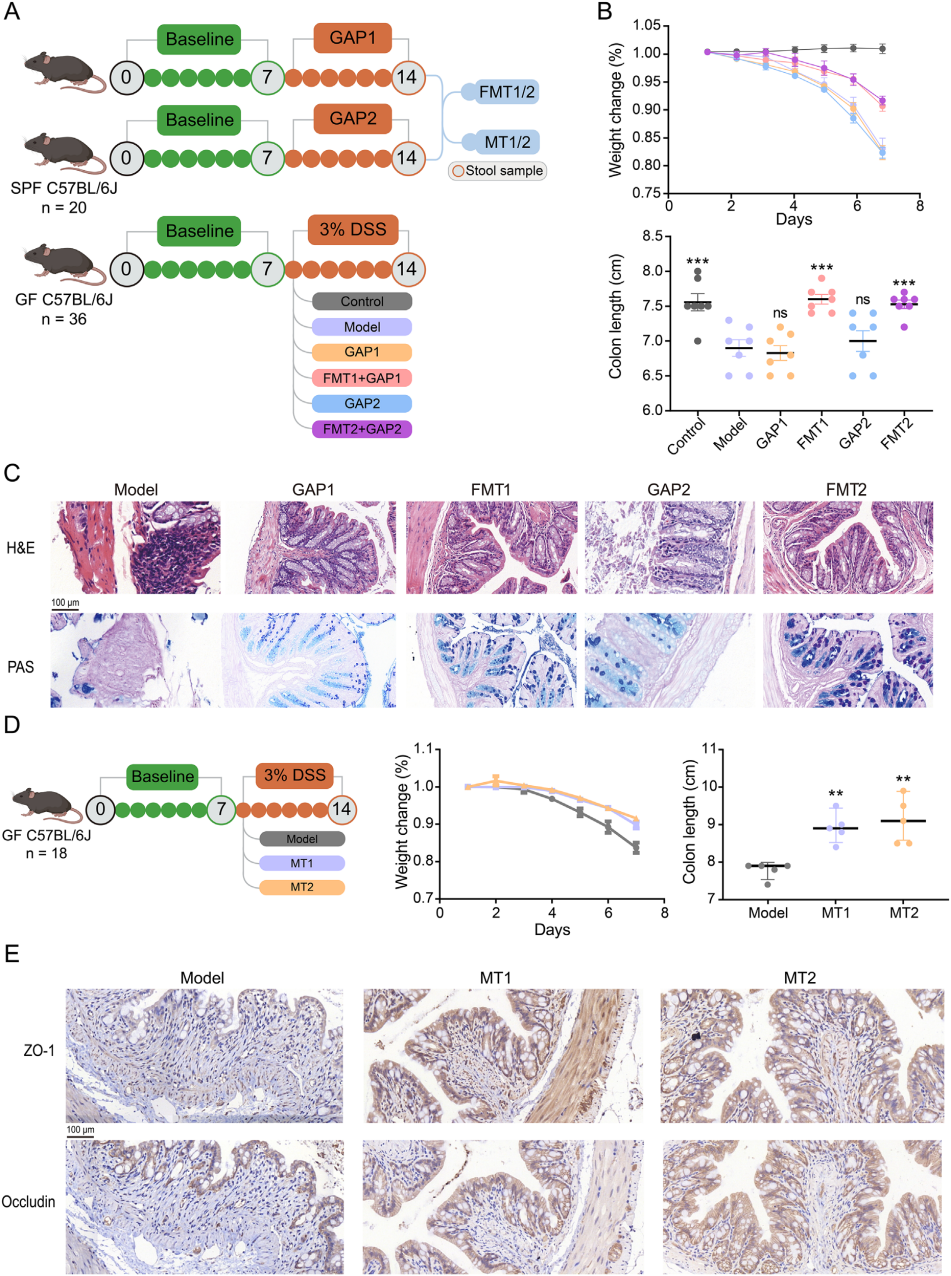
The health benefits of GAP1 and GAP2 depend on the gut microbiota. (A) GAP1 and GAP2 were evaluated for microbiota‐dependent activity using germ‐free mice and fecal microbiota transplantation (FMT). (B) Changes in body weight and colon length of DSS‐treated mice after fecal microbiota transplantation (*n* = 7, per group). (C) H&E staining and PAS staining of the colon in DSS‐treated mice after fecal microbiota transplantation. (D) The efficacy of metabolites derived from GAP1 and GAP2 was evaluated in germ‐free mice after gut microbiota‐mediated metabolism. Changes in body weight and colon length of DSS‐induced mice were evaluated during the remission phase (n = 5 per group). (E) Effects of microbiota‐metabolized GAP1 and GAP2 on ZO‐1 and occludin in colon immunohistochemistry of DSS‐treated mice during the remission phase of colitis. One‐way analysis of variance (ANOVA) combined with Tukey's test was used for gene expression analysis. “ns” indicates *p* > 0.05; ^**^ indicates *p* < 0.01 and ^***^ indicates *p* < 0.001.

Furthermore, in the germ‐free mouse model, we found that gavage with fecal metabolites from mice treated with GAP1 or GAP2 alleviated the weight loss caused by DSS‐induced colitis and significantly reduced colon shortening (Figure 3D). H&E and PAS staining showed that compared with the model group, mice treated with gavage of GAP1 or GAP2 fecal metabolites exhibited an intact intestinal epithelial structure, reduced connective tissue hyperplasia, decreased focal LPL, and an increased abundance of mucin‐producing cells (blue) (Figure S4C). Immunohistochemical analysis revealed a significant increase in the number of tight junction proteins ZO‐1 and occludin (Figure 3E). Taken together, these results indicate that the beneficial effects of GAP1 and GAP2 on improving intestinal inflammation and protecting the intestinal barrier depend on gut microbiota and their metabolites.

### 2.4 GAP1 And GAP2 Show Differential Repair Effects during the Repair Phase of Intestinal Barrier Injury

To better replicate the clinical scenario of patients with genuine intestinal barrier dysfunction, we initiated the polysaccharide intervention only after DSS‐induced intestinal barrier damage (Figure 4A). Surprisingly, GAP2 superior a better intestinal barrier repair effect compared to GAP1. GAP2 intervention significantly accelerated the recovery of body weight and colon length in the DSS‐induced colitis model (Figure 4B,C). Histopathological analysis revealed that intestinal glands in the lamina propria of the GAP1 group still disappeared, accompanied by substantial infiltration of lymphocytes and neutrophils. In contrast, the intestinal epithelial mucosal layer in the GAP2 group maintained an intact structure, characterized by well‐preserved epithelial architecture with tightly arranged cells, an increased number of intestinal glands within the lamina propria, and reduced focal infiltration of LPLs. Compared to the model and GAP1 groups, the GAP2 group showed significantly elevated mucin levels and mucin‐secreting cells (blue staining), thereby facilitating mucosal repair during colitis recovery (Figure 4D,E). Colon immunohistochemistry demonstrated that, relative to the model and GAP1 groups, GAP2 enhanced both the abundance of ZO‐1 and occludin–producing cells and the expression of these tight junction proteins (Figure 4F). Polysaccharide structural analysis indicated that GAP1 and GAP2 had comparable average molecular weights; and the difference in their intestinal barrier repair effects may be attributed to differential regulation of intestinal microorganisms and their metabolites induced by component differences [28]. These results demonstrated that GAP2 more effectively promotes the repair of intestinal barrier injury in colitis.

**FIGURE 4:**
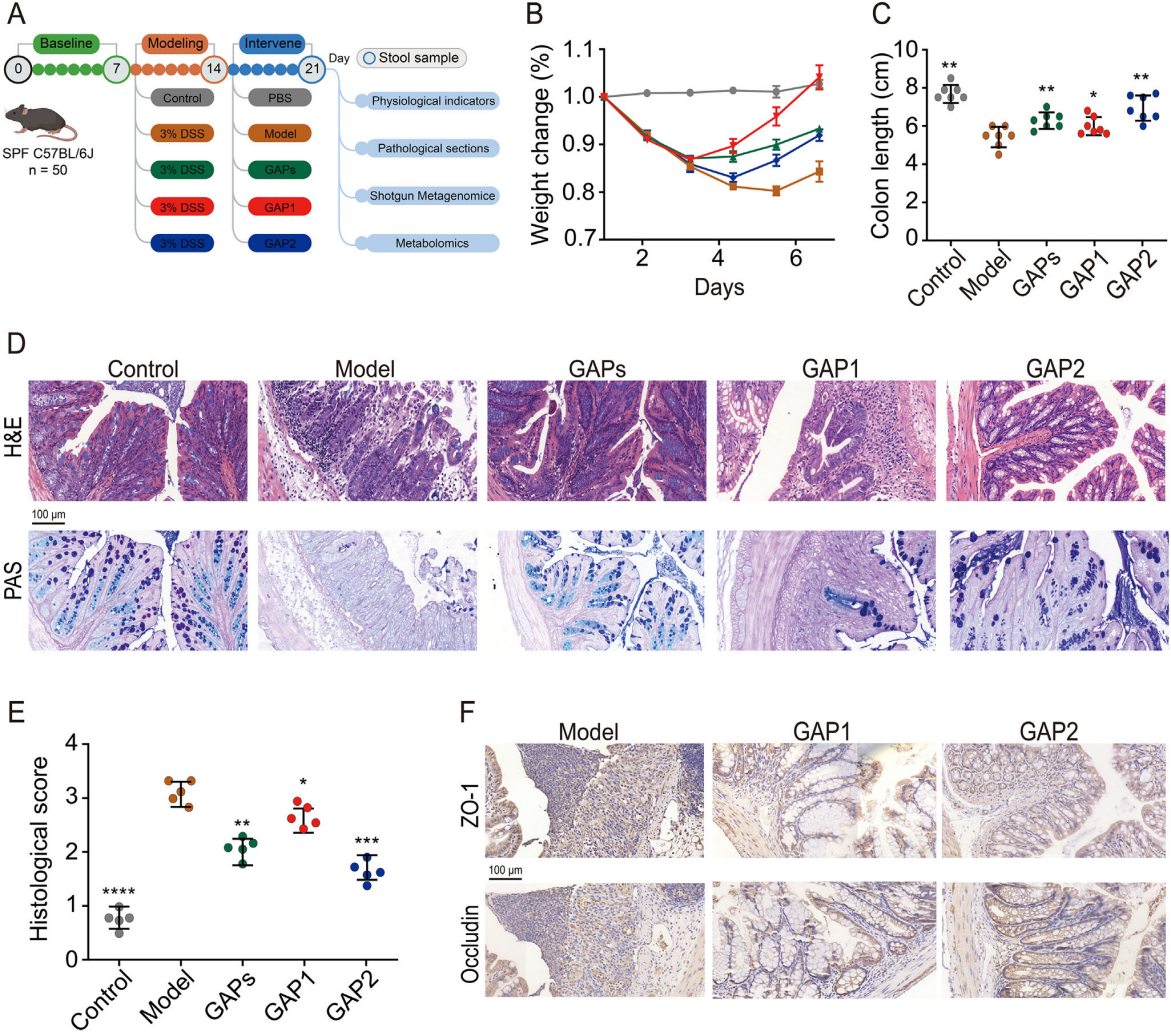
Efficacy of GAP1 and GAP2 in the repair phase of DSS‐induced intestinal barrier damage. (A) Experimental design of the mechanism of action of GAP1 and GAP2 in the repair phase of intestinal barrier damage. (B, C) Improvements of GAP1 and GAP2 on body weight and colon length in DSS‐treated mice during the damage repair phase (*n* = 7, per group). (D,E) H&E and PAS staining of the colon and section scores of DSS‐treated mice after GAP1 and GAP2 treatment during the damage repair phase. (F) Effects of GAP1 and GAP2 on immunohistochemistry of ZO‐1 and Occludin in the colon of DSS‐treated mice during the damage repair phase (*n* = 5, per group). One‐way analysis of variance (ANOVA) combined with Tukey's test was used for gene expression analysis. “ns” indicates *p* > 0.05; ^*^ indicates *p* < 0.05; ^**^ indicates *p* < 0.01; ^***^ indicates *p* < 0.001 and ^****^ indicates *p* < 0.0001.

### 2.5 GAP2 Significantly Enriches *P. vulgatus* and Tridecylic Acid Promotes Intestinal Barrier Repair

Although both GAP1 and GAP2 demonstrated protective effects against DSS‐induced intestinal barrier injury in the initial screening, GAP2 exhibited a more pronounced beneficial effect than GAP1 during the injury repair phase. Therefore, subsequent mechanistic studies focused on GAP2. To identify the key effector bacteria and metabolites involved in GAP2‐mediated intestinal barrier repair, multi‐omics analysis was performed on mouse fecal samples [29]. Shotgun metagenomic sequencing revealed a significant recovery of α‐diversity. Venn diagram and principal coordinate analysis (PCoA) results showed that the microbial community composition in the GAP2 group differed markedly from that of the model group (Figure 5A–C). Furthermore, genus‐level compositional analysis indicated that GAP2 enriched *Bacteroides*, *Parabacteroides*, unclassified *Oscillospiraceae*, unclassified *Lachnospiraceae*, *Akkermansia*, and *Alistipes* (Figure 5D). LEfSe and support vector machine analyses identified *P. vulgatus* as a key bacterium enriched by GAP2 during intestinal barrier injury repair (Figure 5E,F). KEGG pathway enrichment analysis revealed that GAP2 primarily promoted fatty acid biosynthesis, pimeloyl‐ACP biosynthesis from decanoyl and other precursors, and the reductive acetyl‐CoA pathway (Figure 5G). Meanwhile, it upregulated TonB‐dependent starch‐binding outer membrane protein SusC, α‐glucosidase, unsaturated rhamnoglucan hydrolase, xylan 1,4‐β‐diglucosidase, DNA repair proteins SbcC/Rad50, etc (Figure S5A).

**FIGURE 5:**
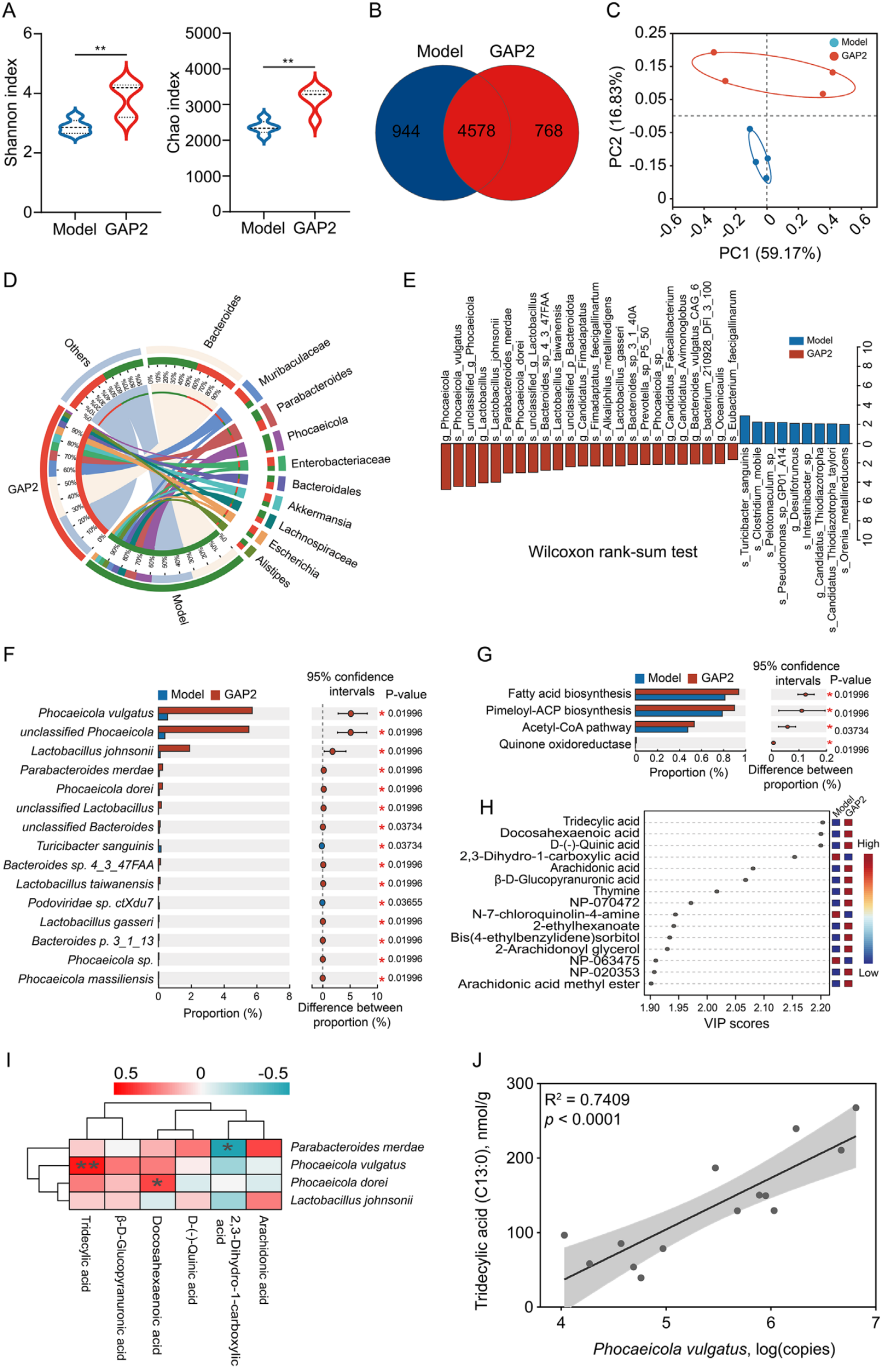
Analysis of key effector bacteria and material basis for GAP2 improving intestinal barrier dysfunction. (A) Alpha diversity boxplot in colon (*n* = 4, per group). (B) Venn analysis. (C) PCoA analysis (*n* = 4, per group). (D) Species abundance composition analysis. (E) LEfSe analysis in different microbiota composition. (F) Species and VFDB contribution analysis (top 15). (G) KEGG pathway enrichment analysis between model and GAP2. (H) Metabolites variance analysis in GAP2 and Model (*n* = 4, per group). (I) Spearman's two‐tailed correlation analysis between differential species and representative fecal metabolites. (J) Linear regression analysis between tridecylic acid and the absolute abundance of *P. vulgatus* in mouse feces after GAP2 treatment. One‐way analysis of variance (ANOVA) combined with Tukey's test was used for gene expression analysis. “ns” indicates *p* > 0.05; ^*^ indicates *p* < 0.05 and ^**^ indicates *p* < 0.01.

Metabolic pathway and functional annotation analyses showed that GAP2 primarily enhanced the metabolic activity of pathways such as glutathione metabolism, glycine‐serine‐threonine family amino acid metabolism, and pantothenate/coenzyme. Biosynthesis was accompanied by a decrease in the relative expression of Type IV pili (VF0082), capsule (CVF186), and lipopolysaccharide (LPS, VF0561) (Figure S5B,C). Notably, untargeted metabolomics identified tridecylic acid as a key metabolite significantly enriched by GAP2 during intestinal barrier repair (Figure 5H). Among all species and fatty acids with differential enrichment, only tridecylic acid was significantly associated with increased *P. vulgatus* levels in DSS‐treated mice supplemented with GAP2 (Figure 5I). Furthermore, in recipient mice supplemented with GAP2, there was a positive correlation between the fecal tridecylic acid concentration and the absolute copy number of *P. vulgatus* (Figure 5J).

### 2.6 Evidence for a Causal Role of GAP2 in Enrichment of *P. vulgatus* and Induction of Tridecylic Acid Production Tridecylic Acid

Taken together, the metagenomic and metabolomic data indicate a GAP2‐associated increase in *P. vulgatus* abundance and a concurrent enrichment of the postbiotic metabolite tridecylic acid (Figure 5). However, these observations are correlational and cannot, by themselves, determine whether GAP2 directly drives *P. vulgatus* expansion and tridecylic acid production. Therefore, we next performed mechanistic experiments in isolated cultures and functional genomics to test whether GAP2 promotes *P. vulgatus* growth and induces tridecylic acid biosynthesis. Bioactive dietary polysaccharides can actively regulate the host intestinal microbiota, and different structural types exert selective regulatory effects on specific microbial taxa [30, 31]. Supplementation with inulin, xylan, arabinoxylan, mannan, and β‐glucan may increase the abundance of *P. vulgatus* in the intestine. We performed pure strain culture experiments using a single carbon source in vitro to evaluate the targeted regulatory effect of GAP2 on *P. vulgatus*. At 24 h of culture, the promoting effect of GAP2 on *P. vulgatus* was similar to that of glucose, with an OD value of 0.75, which was significantly higher than that of other dietary polysaccharides (Figure 6A). Furthermore, qPCR analysis showed that compared with GAP1, GAP2 increased the absolute amount of *P. vulgatus* to 10^9^ CFU/mL, representing a 10.21‐fold increase (Figure 6B). Meanwhile, the level of tridecylic acid in the culture supernatant of *P. vulgatus* increased. Importantly, tridecylic acid levels varied significantly across different growth substrates, with GAP2 resulting in the highest yield (Figure 6C). Although glucose can promote the tridecylic acid production in *P. vulgatus*, its effect is not specific within the complex gut environment.

**FIGURE 6:**
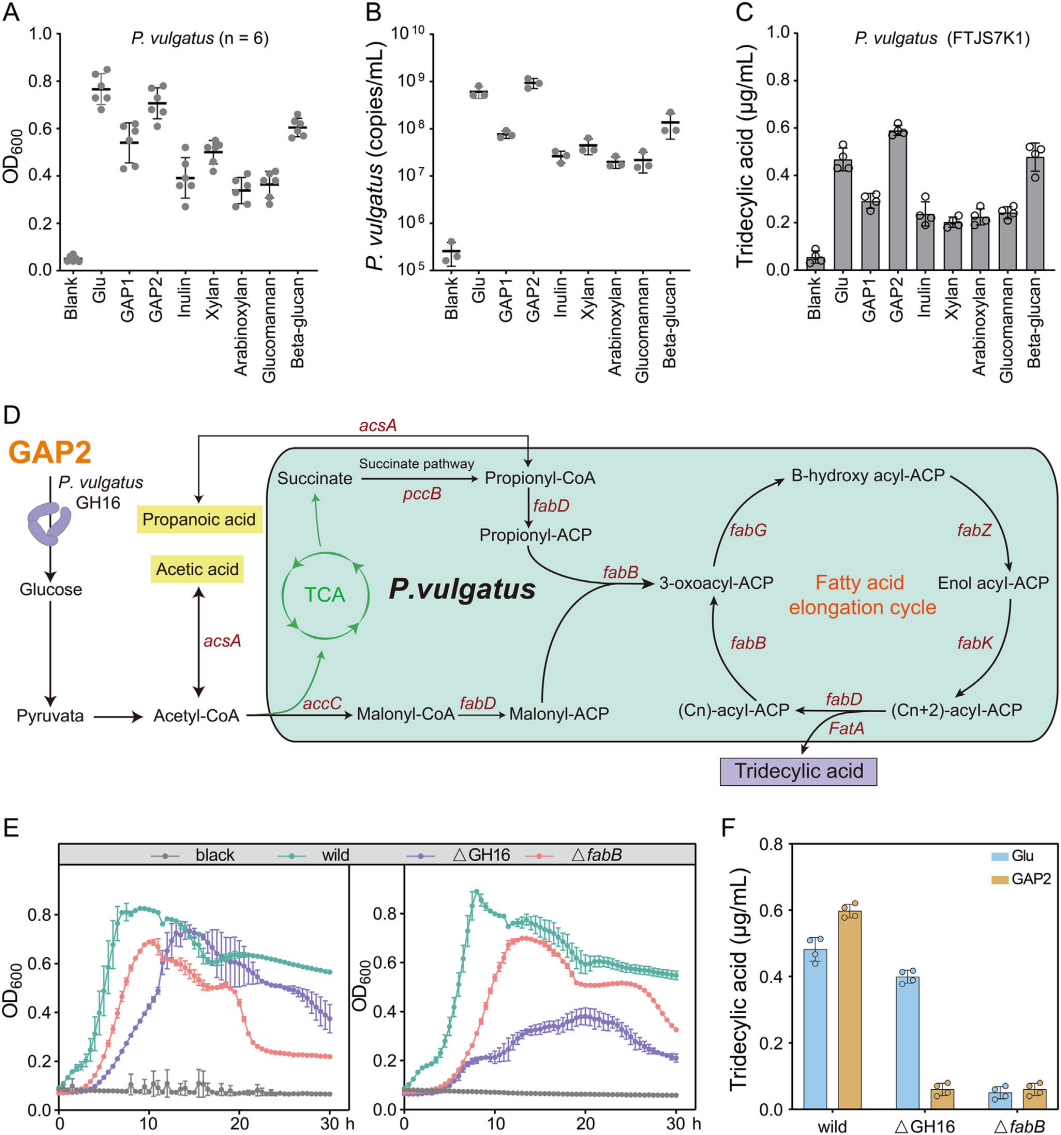
*P. vulgatus* synthesizes tridecylic acid to improve intestinal barrier dysfunction. (A,B) Regulatory effects of *G. applanatum* active polysaccharides and common dietary polysaccharides on *P. vulgatus*. (C) Concentration of tridecylic acid in the culture supernatant of *P. vulgatus* after intervention with *G. applanatum* active polysaccharides and common dietary polysaccharides (*n* = 3, per group). (D) Metabolic pathway of tridecylic acid in *P. vulgatus*. (E) Growth curves of *P. vulgatus* mutants in a medium with Glu and GAP2 as sole carbon sources (*n* = 4, per group). (F) Concentration of tridecylic acid in the culture supernatant of *P. vulgatus* mutants grown in GAP2 (*n* = 4, per group).

To directly confirm whether *P. vulgatus* can synthesize tridecylic acid, we performed whole‐genome sequencing followed by COG‐based pathway annotation, which revealed the presence of long‐chain fatty acid biosynthesis pathways. Meanwhile, CAZymes analysis indicated that *P. vulgatus* harbors the key hydrolase GH16, responsible for GAP2 degradation (Figure 6D; Figure S6). Notably, all essential genes involved in tridecylic acid biosynthesis were detected (Figure S7), indicating that *P. vulgatus* is capable of producing tridecylic acid. To further investigate the utilization of GAP2 by *P. vulgatus* and its ability to synthesize tridecylic acid, we constructed gene deletion mutants of key genes using the CRISPR‐Cas system [32]. Under GAP2 culture conditions, knockout of the key CAZymes gene GH16 significantly inhibited the growth of *P. vulgatus*. *FabB* typically corresponds to key enzymatic steps involved in fatty acid elongation and/or synthesis. In contrast, knockout of the *fabB* gene did not significantly affect the growth rate but blocked the synthesis of tridecylic acid by *P. vulgatus* (Figure 6E,F).

### 2.7 Tridecylic Acid Repairs Intestinal Barrier Dysfunction by Modulating the Rho/CDC42 Signaling Pathway

To elucidate the mechanisms by which GAP2 improves intestinal barrier function—specifically through targeting *P. vulgatus* and promoting the production of the postbiotic tridecylic acid—we performed a transcriptomic analysis of colon tissue. Gene volcano plot analysis revealed that, following GAP2 treatment, 228 genes were downregulated and 67 genes were upregulated (Figure 7A). The heatmap of the top 30 enriched genes showed consistent clustering within the same group, indicating minimal inter‐group differences and supporting the validity of subsequent analyses (Figure S8). Pathway enrichment analysis revealed that GAP2 primarily influenced the cell adhesion network and stress response by regulating integrin‐mediated intercellular adhesion and the response to unfolded and topologically misfolded proteins, involving genes such as CDC42, Rho, BAIAP2, and Par3 (Figure 7B). Meanwhile, GAP2 reduced the regulation of purine nucleotide metabolism, nucleotide metabolism, small molecule metabolism, precursor metabolite production, energy regulation pathways, and ligase activity regulation (Figure S9A,B).

**FIGURE 7:**
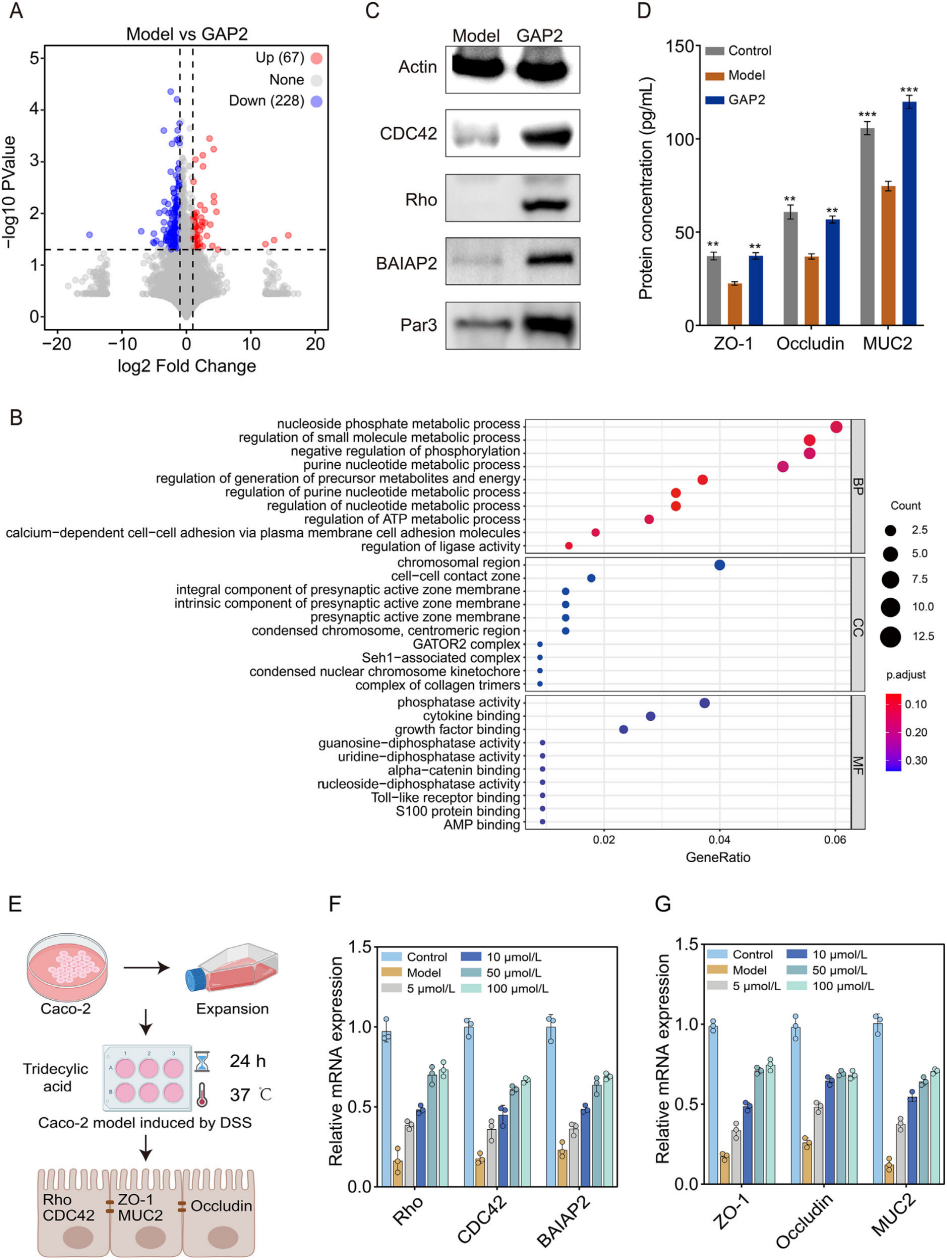
Mechanism of action of GAP2 in improving intestinal barrier damage. (A) Volcano plot of genes from transcriptome analysis of mouse colon under GAP2 treatment. (B) GO enrichment analysis and pathway enrichment analysis of upregulated genes in the feces of DSS‐induced colitis mice administered with GAP2 by gavage (n = 6 per group). (C) Analysis of the expression levels of intestinal barrier repair genes and the expression levels of tight junction proteins in the colon of mice treated with GAP2. (D) Effects of GAP1 and GAP2 on the content of intestinal barrier‐related proteins (*n* = 6, per group). (E–G) Study design, relative expression levels of intestinal barrier repair‐related enzymes and tight junction proteins in DSS‐induced Caco‐2 cells (with or without tridecylic acid treatment) (n = 3, per group). ^*^ Indicates *p* < 0.05; ^**^ indicates *p* < 0.01, ^***^ indicates *p* < 0.001.

Western blot experiment showed that GAP2 increased the levels of CDC42, Rho, BAIAP2, and Par3, accompanied by elevated levels of tight‐junction proteins (Figure 7C). Compared with the model group, GAP2 administration increased the concentrations of intestinal epithelial Occludin and ZO‐1, and the concentration of MUC2 also increased significantly (Figure 7D). In addition, tridecylic acid restored the expression of Rho, CDC42, and BAIAP2 genes related to intestinal barrier maintenance in DSS‐induced Caco‐2 cells and markedly increased the levels of tight junction proteins, suggesting that tridecylic acid may contribute to intestinal barrier protection through modulation of the Rho/CDC42 pathway (Figure 7E–G).

### 2.8 In Patients with Intestinal Barrier Damage, the Abundance of *P. vulgatus* and Genes in the Tridecylic Acid Biosynthetic Pathway are Reduced

To investigate the role of *P. vulgatus* and its tridecylic acid synthesis‐related genes in intestinal inflammation and barrier dysfunction, we conducted a systematic comparison of metagenomic data from UC patients and healthy individuals [33] (Figure 8A). The results revealed a significant reduction in microbial diversity in the UC group, with community composition differing notably from that of healthy controls. Both Shannon and Chao1 indices were significantly lower, and principal coordinate (PCoA) analysis demonstrated a clear separation between the UC and healthy groups (*p*<0.05) (Figure 8B,C), indicating that the intestinal microbial structure was disrupted and shifted to a low‐diversity state in the UC. Further characterization showed that the healthy group was enriched with typical bacteria such as *P. vulgatus*, *Bacteroides intestinalis*, and *Bacteroides faecis*, while the UC group was dominated by significantly characteristic bacteria including *Monas*, *Cryptococcus faecalis*, *Bacillus lacticus*, and *Longicatena caecimuris*. Among them, the abundance of *P. vulgatus* decreased most significantly in UC, making it a key marker for distinguishing the two groups (Figure 8D). Functional annotation of clinical metagenomic data revealed that key genes related to tridecylic acid synthesis, including *fabB*, *fabG*, *fabZ*, *fabK*, and *FatA*, were markedly reduced in the UC group. Combined with comprehensive analysis of the whole genome of *P. vulgatus* and the fatty acid synthesis pathways, these results suggest that this bacterium and its product tridecylic acid have potential protective effects in maintaining and repairing intestinal barrier function (Figure 8E); In other words, inhibition of this metabolic pathway in the UC may be associated with impaired barrier integrity and exacerbated inflammation.

**FIGURE 8:**
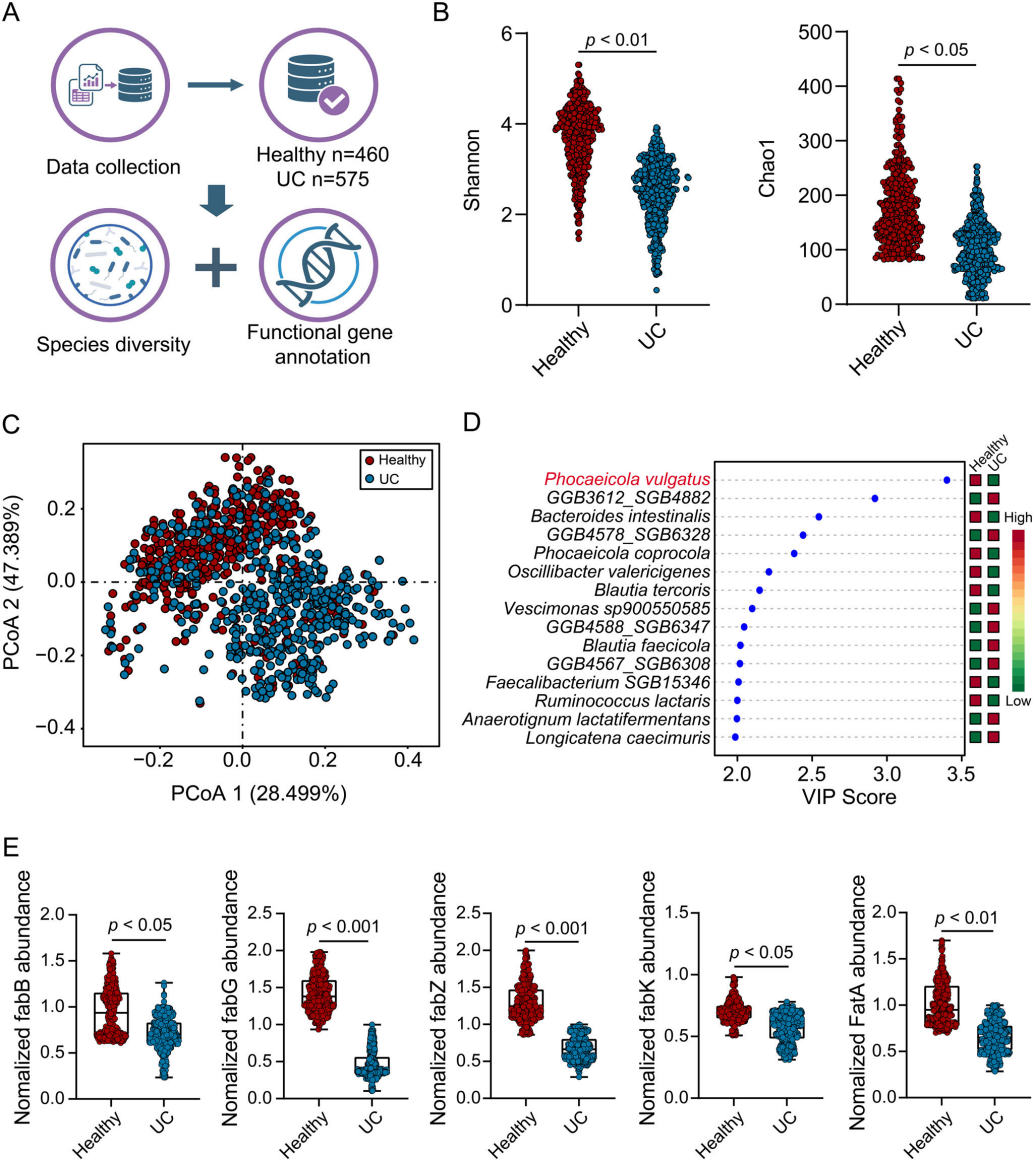
The role of *P. vulgatus* and tridecylic acid in UC. (A) Flowchart of fecal metagenome analysis in UC clinical cohorts. (B) α diversity represented by Shannon index and Chao1 index (*n* = 460, per healthy group; *n* = 575, per UC group). (C) Principal coordinate analysis (PCoA) of all samples based on weighted UniFrac distance (ANOSIM test) (*n* = 460, per healthy group; *n* = 575, per UC group). (D) Variable importance in PLS‐DA prediction (VIP) scores showing the ability of different taxa to distinguish groups. Taxa with VIP scores > 1.5 are considered very important in discrimination (*n* = 460, per healthy group; *n* = 575, per UC group). (E) Relative abundance (percentage) of key bacterial genes involved in tridecylic acid synthesis based on shotgun metagenomic analysis (*n* = 460, per healthy group; *n* = 575, per UC group). One‐way analysis of variance (ANOVA) combined with Tukey's test was used for gene expression analysis.

These results collectively indicate a central conclusion: the gut microbiota of healthy individuals maintains barrier homeostasis by enriching *P. vulgatus* and its fatty acid synthesis pathway. In contrast, the attenuation of this pathway in UC patients is associated with barrier dysfunction and inflammatory, providing a theoretical foundation for intervention strategies targeting microbial metabolites.

## 3 Discussion

Ulcerative colitis (UC) has emerged as a significant public health concern due to its increasing incidence [34, 35, 36]. Multiple factors, including intestinal barrier dysfunction, abnormal immune responses, and gut microbiota dysbiosis, are believed to be closely associated with the pathogenesis of UC [37, 38]. Dietary intervention aimed at improving intestinal barrier dysfunction, is considered a promising therapeutic strategy [39, 40]. In this study, we identify GAP2 as an active component capable of alleviating UC‐associated barrier dysfunction in a gut‐ecosystem–dependent manner. Mechanistically, GAP2 modulates the gut microbiota by enriching *P. vulgatus* and shifts microbial metabolic output toward the production of the postbiotic tridecylic acid. Conceptually, these findings support a microbe–metabolite axis framework: a defined microbial niche acts as an upstream determinant that generates a specific metabolite, which in turn engages downstream host signaling pathways to influence intestinal barrier integrity. By focusing on the axis (*P. vulgatus—*tridecylic acid—barrier‐related signaling), this work provides a more interpretable and testable model for postbiotic‐inducing dietary interventions and offers a rationale for further validation of targeting this microbe–metabolite–host interface in intestinal inflammatory disorders.

We further discovered that UC patients exhibited reduced levels of *P. vulgatus* and a loss of key genes required for tridecylic acid synthesis, underscoring the important role of *P. vulgatus* in UC pathogenesis. *P. vulgatus*, a common commensal bacterium in the human gut, primarily colonizes the colon and is present in up to 90% of the global population [41, 42, 43]. It contributes to gut health by decomposing complex plant carbohydrates and dietary fibers to produce metabolites such as SCFAs [44]. This bacterium is not only crucial for maintaining intestinal health but also possesses various probiotic potentials and is associated with multiple diseases, including obesity, cardiovascular diseases, type 2 diabetes, intestinal inflammation, and endothelial dysfunction [28, 45, 46, 47]. *P. vulgatus* can produce pantothenate (vitamin B5), promote glucagon‐like peptide‐1 secretion, and activate the release of hepatic fibroblast growth factor 21 (FGF21), thereby acting on hypothalamic neural circuits to suppress carbohydrate intake desire and modulate host inflammatory symptoms [48]. Research has also found that *P. vulgatus* can elevate SCFAs levels, reduce LPS‐induced cytokine mRNA changes in colonic tissue, and influence the ratio of regulatory T cells in mesenteric lymph nodes [28]. Differences across studies in strain‐specific functions, host immune and barrier status, and the available substrates or metabolic environment could lead to divergent outcomes [46, 49]. Accumulating evidence shows that structurally distinct polysaccharides selectively promote the growth of specific bacterial strains, largely due to differences in their backbone and side‐chain structures; side chains generally reduce polysaccharide degradability and bioavailability [50, 51]. Structural characterization indicated that, compared with GAP1, GAP2 lacks side‐chain structures and is a β‐glucan, thereby exhibiting higher bioavailability and serving as the preferential carbon source for *P. vulgatus*. Moreover, its exopolysaccharides have been reported to restore mucosal barrier integrity and rebalance the gut microbiota in DSS‐induced UC [52]. Together, these findings support the protective role of *P. vulgatus* in maintaining gut health, especially in the context of UC.

Fatty acids perform diverse and essential roles in energy metabolism, membrane structure, and signal transduction within intestinal epithelial cells, thereby influencing mucosal barrier function and inflammatory responses [53, 54]. As energy substrates, fatty acids undergo β‐oxidation to supply energy for epithelial cells, supporting mucus layer synthesis and the expression of tight junction proteins, which enhances barrier function [52, 55]. Their metabolites also modify membrane lipid composition, affecting membrane fluidity and receptor clustering, and modulating the threshold and kinetics of inflammatory signals [56]. Fatty acids and their metabolites regulate the expression of inflammatory genes through nuclear receptors — such as peroxisome proliferator‐activated receptors (PPARs), liver X receptors (LXRs), farnesoid X receptor (FXR) —as well as transcription factors (nuclear factor‐κB (NF‐κB), activator protein‐1 (AP‐1), signal transducers and activators of transcription (STATs) [57, 58]. Previous studies have demonstrated that metabolic alterations in medium‐ and long‐chain fatty acids are closely associated with the onset and progression of UC [52]. Our earlier research similarly indicated that hyaluronic acid can promote the proliferation of *Bacteroides thetaiotaomicron* and *Bacteroides caccae*, elevates myristic acid levels in intestinal, and reduces LPS‐induced host inflammatory responses [59]. Recent studies have shown that combining galacto‑oligosaccharides (GOS) with *Limosilactobacillus reuteri* stimulates *Bacteroides acidifaciens* to generate pentadecanoic acid, which inhibits NF‐κB activation and enhances intestinal tight junctions [30]. Consistently, our study observed a marked reduction of the odd‐chain fatty acid tridecylic acid in the intestines of DSS‐treated mice. Additionally, meta‐analysis of clinical UC cohorts revealed a significant decline in the abundance of genes involved in tridecylic acid synthesis. Previous research has demonstrated that tridecylic acid exerts anti‐inflammatory, anti‐fibrotic, and anti‐cancer effects through multiple pathways, including activation of AMP‐activated protein kinase (AMPK) and peroxisome proliferator‐activated receptor alpha/delta (PPAR‐α/δ), and inhibition of mechanistic target of rapamycin (mTOR), janus kinase 3 – signal transducer and activator of transcription 1 (JAK3–STAT1), and histone deacetylase 6 (HDAC6), in both in vitro and in vivo models [60, 61, 62]. However, the mechanisms by which tridecylic acid inhibits inflammation and improves barrier dysfunction remain unclear. Rho is a critical regulatory protein involved in stabilizing the perijunctional actin‐myosin cytoskeleton and acts as a signaling molecule that promotes the deposition of ZO‐1 and occludin at tight junctions, thereby maintaining intestinal barrier function [63]. Our research demonstrated that tridecylic acid intervention effectively activates the Rho and CDC42 signaling pathways, increasing the expression of MUC2, ZO‐1, and occludin, and promoting the restoration of epithelial barrier integrity.

Current research on tridecylic acid primarily focuses on its role in the host's fatty acid metabolism, where it is closely linked to conditions such as metabolic syndrome, non‑alcoholic fatty liver disease, cardiovascular diseases, depressive symptoms following stroke and UC [64]. Additionally, tridecylic acid exhibits antimicrobial properties, inhibiting the growth of pathogenic bacteria and fungi [65]. It also functions as an anti‑adhesive and anti‑biofilm agent, suppressing *Escherichia coli* persistence and biofilm formation, which is significant in studies of bacterial infections [66]. Research on its impact on host physiological health remains in the preliminary stages. Although initial studies suggest that tridecylic acid plays a role in the host fatty acid metabolic network [67], the direct causal relationship and clinical significance of tridecylic acid for host physiological health are still under investigation. Future research involving clinical population studies, intervention trials, and mechanistic experiments is necessary to clarify its physiological functions and potential therapeutic applications.

It has been reported that gut microbiota possess a broad capacity to synthesize long‐chain fatty acids, such as members of the genera *Bacteroides*, *Bifidobacterium*, and *Lactobacillus* [68, 69, 70]. A recent study demonstrated that *Parabacteroides distasonis* can produce pentadecanoic acid (C15:0) from inulin, thereby alleviating non‑alcoholic steatohepatitis [30]. Additionally, other research has shown that *Bacteroides acidifaciens* utilizes acetate and propionate generated from GOS and *Limosilactobacillus reuteri* to synthesize C15:0, which cooperatively mitigates intestinal inflammation and barrier dysfunction [5]. Our earlier work also found that dietary hyaluronic acid specifically modulates *Bacteroide thetaiotaomicron* and *Bacteroide caccae*, promoting the synthesis of myristic acid (C14:0) [59]. This study further demonstrates that GAP2 specifically stimulates the growth of *P. vulgatus* while elevating tridecylic acid levels. Regarding the biosynthesis of long‐chain fatty acids, most current research focuses on C15:0; however, tridecylic acid also represents a significant fatty acid product of gut microbiota, particularly within the genus *Bacteroides*, and holds potential physiological and metabolic relevance. Long‐chain fatty acid biosynthesis is initiated by the incorporation of external three‐carbon units, such as propionyl‐CoA and propionyl‐ACP, which act as precursor substrates [71]. Moreover, all enzymes required for tridecylic acid biosynthesis—including β‑ketoacyl‑ACP synthase II (FabF), β‑ketoacyl‑ACP reductase (FabG), β‑hydroxyacyl‑ACP dehydratase (FabA), enoyl‑ACP reductase (FabK), malonyl‑CoA:ACP transacylase (FabD), and aliphatic acyl‑CoA thioesterase (PaaL/FATA)—are encoded within the genome of *P. vulgatus* [70]. Therefore, through genetic enhancement engineering and targeted regulatory switch technologies, precise in vivo control over tridecylic acid production by *P. vulgatus* can be achieved, thereby harnessing its potential probiotic benefits. Similarly, recent studies have applied genetic engineering to *P. vulgatus* to enable oxalate degradation and to use porphyran as an exclusive nutrient source for regulating intestinal colonization, providing a conditionally attenuating, reversibly colonizing mechanism as a potential microbial therapeutic strategy for treating enteric hyperoxaluria [72].

Although this study, based on transcriptomic analysis and protein‐level measurements, identified an association between GAP2/tridecylic acid and the Rho/CDC42‐associated signaling network as well as tight junction–related markers, the precise mechanism by which tridecylic acid activates the Rho/CDC42 signaling cascade remains unclear. Future research will aim to elucidate the causal relationship within this pathway by conducting tridecylic acid–induced Rho/CDC42 activation assays, alongside inhibition or knockdown of key upstream regulators and pathway‐blocking experiments. Additionally, human metagenomic data are observational and lack direct measurements of specific metabolites (e.g., tridecylic acid) and downstream signaling pathways (e.g., Rho/CDC42). Consequently, the current conclusions are primarily inferred from mechanistic studies in animal models and in vitro systems, and extrapolation to humans requires further validation. Future investigations should include mechanistic studies in human systems, incorporating metabolomics and metabolite‐level measurements, stratified association analyses across the microbe–metabolite–host axis, and rigorously designed intervention studies to establish causality.

## 4 Conclusions

In summary, our findings demonstrate that GAPs can improve intestinal barrier dysfunction by modulating the metabolic synthesis pathways of the gut microbiota. We established a direct link among dietary GAP2, *P. vulgatus* (a targeted gut commensal with high activity), and tridecylic acid (a postbiotic derived from this bacterium). The activation of key signaling pathways involved in intestinal barrier repair by *P. vulgatus* and tridecylic acid highlights the beneficial role of GAP in maintaining intestinal barrier integrity. Future studies should further evaluate the therapeutic potential of GAP2 for intestinal barrier dysfunction at the clinical level, thereby providing a scientific basis for dietary intervention strategies targeting intestinal barrier‐related disorders.

## 5 Experimental Section

### 5.1 Preparation of GAP1 and GAP2

GAPs were extracted from dry fruiting bodies obtained from Changbai Mountain in Jilin Province, China. These fruiting bodies were identified as *G. applanatum* by the Institute of Microbiology, Chinese Academy of Sciences, and the specimens CCFM20221205 was preserved in the Culture Collections of Food Microbiology (CCFM), Jiangnan University (Wuxi, China). GAPs were produced through hot water extraction followed by ethanol precipitation [25]. Purification was carried out using a HiTrap DEAE FF anion‐exchange column, followed by size‐exclusion chromatography on a HiLoad 16/20 Superdex 75 pg column. The target fractions, GAP1 and GAP2, were then concentrated and lyophilized to obtain purified polysaccharide samples.

### 5.2 Chemical Analysis of GAP1 and GAP2

The monosaccharide compositions of GAP1 and GAP2 were determined using previously established methods. Briefly, the polysaccharide samples were hydrolyzed with trifluoroacetic acid (TFA) and subsequently derivatized with PMP at 70°C for 30 min. The resulting monosaccharide profiles were analyzed using the PMP‐HPIC method on a Thermo Scientific Dionex ICS‐5000 + SP‐5 system, as described previously. FT‐IR spectra were recorded on a NEXUS spectrometer over the wavenumber range of 4000–400 cm^−^^1^. For NMR analysis, samples were dissolved in deuterated water, and both one‐dimensional (^1^H, ^1^ ^3^C) and selected two‐dimensional spectra (HSQC, HMBC, COSY) were acquired on a 400 MHz spectrometer to elucidate glycosidic linkages and structural characteristics [73].

### 5.3 Animal Experiments

All animal experiments were conducted in compliance with the National Research Council's Guide for the Care and Use of Laboratory Animals and were approved by the Animal Ethics Committee of Jiangnan University (Approval Nos. JN. No. 20210615c2000901[168] and JN. No. 20220315c0360501[090]). All procedures strictly followed the approved experimental protocols. Before the initiation of experiments, animals underwent a 1‐week acclimation period under controlled conditions, with food and water available ad libitum, after the acclimation period, all mice were randomly assigned to different experimental groups.

**Animal Experiment 1**: Male C57BL/6J mice (6–8 weeks, 18–20 g; n = 70) were housed under SPF conditions and randomly assigned to 7 groups (n = 10/group): Control, drinking water (days 1–7) then sterile PBS gavage (days 7–14); DSS recovery, drinking water (days 1–7) then 3% DSS in drinking water + PBS gavage (days 7–14); GAPs, 3% DSS (days 7–14) + *Ganoderma applanatum* crude polysaccharides (GAPs, 300 mg/kg, gavage); GAP1–GAP4, 3% DSS (days 7–14) + *G. applanatum* polysaccharide fractions (GAP1, GAP2, GAP3, or GAP4; 50 mg/kg, gavage). Body weight, stool consistency, and fecal occult blood were monitored daily and DAI was recorded. On day 15, mice were euthanized by CO_2_, the intestine was collected, and colon length was measured.
**Animal Experiment 2**: Mice (18–22 g) were randomly assigned to two groups (n = 10 per group): (1) GAP1 and (2) GAP2. The doses of GAP1 and GAP2 were selected based on previous studies. GAP1 group were administered GAP1 (50 mg/kg) suspended in normal saline by oral gavage, and GAP2 group were administered GAP2 (50 mg/kg) suspended in normal saline by the same route. After 7 days of treatment, fecal pellets were collected under sterile conditions as donor material for subsequent fecal microbiota transplantation (FMT).
**Animal Experiment 3**: The fecal microbiota transplantation (FMT) protocol was performed according to previous studies [74]. Six experimental groups were established using germ‐free mice (n = 10 per group) following random allocation: (1) Control, untreated (received regular drinking water and normal saline); (2) Model, 3.0% (w/v) DSS in drinking water for 7 consecutive days plus normal saline; (3) GAP1, DSS + GAP1 (50 mg/kg); (4) FMT1, DSS + FMT prepared from donor feces collected from the GAP1 group in Animal Experiment 2 (0.15 g feces resuspended in 1 mL normal saline); (5) GAP2, DSS + GAP2 (50 mg/kg); and (6) FMT2, DSS + FMT prepared from donor feces collected from the GAP2 group in Animal Experiment 2 (0.15 g feces resuspended in 1 mL normal saline). Body weight, stool consistency, and the presence of fecal blood were recorded daily, and the DAI was evaluated according to previously established criteria [75].
**Animal Experiment 4**: Sterile 3.0% (w/v) DSS drinking water and normal saline were pre‐reduced by incubation in an anaerobic chamber overnight to remove dissolved oxygen. The mice were randomly assigned to three groups (n = 6 per group) and received 3.0% (w/v) DSS in drinking water for seven consecutive days, followed by treatment with (1) model, (2) FMT1, or (3) FMT2. Metabolites were prepared as 0.15 g/ mL feces in normal saline centrifuge at 12000 g for 3 min, and then filter with a 0.22 µL microfiltration membrane. Model group was fed with pre‐reduced normal saline.
**Animal Experiment 5**: Mice (18–22 g) were randomly divided into five groups (n = 10). Except for the control group, all mice received 3.0% DSS for 7 days, followed by a 7‐day recovery period. During recovery, treatments were administered as follows: (1) control (no DSS drinking water), (2) model, (3) GAPs, (4) GAP1, (5) GAP2. During recovery, treatments were administered as follows: control, model, GAPs (300 mg/kg), GAP1 (50 mg/kg), and GAP2 (50 mg/kg). Control and model groups received saline. Clinical parameters were monitored daily.

### 5.4 Enzyme‐Linked Immunosorbent Assay (ELISA) Measurement

The levels of MUC2, ZO‐1, Occludin, COX‐2, iNOS, and MPO in colon tissues were quantified using ELISA. Similarly, IL‐6, IL‐1β, IL‐17, TNF‐α, and IL‐10 were measured according to the manufacturers’ protocols. Frozen colon samples were homogenized in PBS at a 1:9 (w/v) ratio, followed by centrifugation at 12 000 × g for 15 min to obtain a clear supernatant, which was then used for all subsequent analyses [76].

### 5.5 Shotgun Metagenomic Data Analysis

Total genomic DNA was extracted from mouse colon content using the FastDNA Spin Kit for Feces (MP Biomedicals, LLC, Irvine, CA, USA) according to manufacturer's instructions. A paired‐end library was constructed using the NEXTFLEX Rapid DNA‐Seq kit (Bio Scientific, Austin, TX, USA). Paired‐end sequencing was performed on an Illumina NovaSeq 6000 (Illumina Inc., San Diego, CA, USA) at Majorbio Bio‐Pharm Technology Co., Ltd. (Shanghai, China) using NovaSeq Reagent Kits, following the manufacturer's instructions (www.illumina.com). The data were analyzed using the free online platform of the Majorbio Cloud Platform (www.majorbio.com) [77, 78].

### 5.6 Histological Analysis

Colonic tissues from DSS‐treated mice were promptly fixed in 4% paraformaldehyde, paraffin‐embedded, and sectioned at 4–5 µm for H&E staining. Histopathological images were captured using a light microscope, and tissue injury was independently assessed in a blinded manner according to standard scoring criteria. After fixation in Carnoy's solution for 24–48 h, colon tissues were dehydrated and embedded in wax. Specimens were then sectioned at a thickness of 4 µm and stained with Alcian Blue–periodic acid–Schiff. Stained specimens were digitized using a slide scanner (3DHISTECH Ltd, Hungary) for further analysis. CaseViewer 2.4 software was used to examine selected areas at ×400 magnification. Using Image‐Pro Plus 6.0, measurements were taken at five points per section: intestinal villous epithelium length and corresponding cupula cell count. Neutral mucus (purple), acidic mucus (blue), and colon tissue areas were quantified in pixels. The mucus percentage was then calculated from these measurements [79].

### 5.7 Immunohistochemistry, Periodic Acid‐Schiff, and Immunofluorescence Staining

Colon specimens were fixed in 4% formaldehyde, embedded in paraffin, and sectioned for subsequent analyses. Tissue morphology was evaluated by H&E and Schiff staining. In addition, immunohistochemical staining was performed on paraffin sections using antibodies against ZO‐1 and occludin. Representative microscopic fields from each section were then selected for imaging and analysis.

### 5.8 Western Blot Analysis

Total protein was extracted from colon tissues using RIPA buffer supplemented with a protease inhibitor cocktail. Protein samples were separated by SDS‐PAGE and transferred onto polyvinylidene fluoride (PVDF) membranes. After blocking with 5% skim milk, the membranes were incubated overnight at 4°C with primary antibodies targeting β‐actin, SAPK/JNK, NF‐κB p65, and MAPK1. Following washing steps, the membranes were treated with HRP‐conjugated secondary antibodies, and protein bands were subsequently detected using an enhanced chemiluminescence system.

### 5.9 Short‐Chain Fatty Acids (SCFAs) Analysis

SCFAs in colon samples were quantified following a previously described protocol [80]. Briefly, approximately 40 mg of lyophilized material was resuspended in 0.5 mL of saturated NaCl and mechanically disrupted at 65 Hz for 90 s. The mixture was subsequently acidified with 40 µL H_2_SO_4_ and thoroughly vortexed. SCFAs were extracted by adding 1 mL of anhydrous ether, followed by centrifugation at 15 000 × g for 15 min at 4°C. The upper organic layer was collected and subjected to GC–MS analysis.

### 5.10 Transcriptomics Analysis

Total RNA was isolated from mouse colon tissues using TRIzol Reagent, followed by DNase I treatment to eliminate genomic DNA contamination. RNA quality was assessed with an Agilent 2100 Bioanalyzer, and concentration was measured using a NanoDrop ND‐2000 spectrophotometer. Samples meeting predefined quality criteria were used for downstream library construction. Sequencing libraries were prepared from 2 µg of total RNA using the Illumina TruSeq RNA Sample Preparation Kit and subjected to high‐throughput sequencing on an Illumina platform. Differential gene expression analysis was followed by Gene Ontology (GO) enrichment analysis using GOatools based on Fisher's exact test, with multiple testing corrections applied, including the Benjamini–Hochberg procedure. Adjusted *p*‐values ≤ 0.05 were considered statistically significant [81].

### 5.11 Liquid Chromatography‐Mass Spectrometry (LC‐MS) Analysis

Fecal untargeted metabolomics analysis was performed using liquid chromatography‐mass spectrometry (LC‐MS). Fecal samples were stored at −80°C and thawed at 4°C before preparation. A solvent mixture (methanol:acetonitrile:water = 2:2:1) was added at eight times the volume to each fecal sample (50 mg) to extract metabolites. Magnetic beads were added to the fecal solvent and vortexed to mix (65 Hz, 6 times with an interval of 10 s). And then centrifugation at 4°C at 14 000 rpm for 15 min. The supernatants were transferred to a new 1.5 mL centrifuge tube and dried in a centrifugal vacuum evaporator (SPD131DDA, Thermo Scientific, USA), re‐dissolved with 200 µL of mixed solvent (methanol/water  =  4:1), and then centrifuged at 4°C at 14 000 rpm for 15 min [82]. Metabolites were extracted with methanol and analyzed using an UltiMate 3000 UHPLC system coupled to a Q Exactive mass spectrometer equipped with an electrospray ionization source operating in full‐scan MS/dd‐MS^2^ mode. Separation was carried out on an HSS T3 column (2.1 × 100 mm, 1.8 µm) maintained at 40°C. Data processing included feature detection, peak integration, and normalization under both positive and negative ion modes. Multivariate analysis was performed by calculating VIP values with SIMCA 14.1 and constructing OPLS‐DA models to discriminate between groups. Functional interpretation of metabolic alterations was conducted using MetaboAnalyst 5.0 [83].

### 5.12 Caco‐2 Cell Treatment by Tridecylic Acid

Caco‐2 cells were seeded into 6‐well plates at a density of 5 × 10^6^ cells/mL (2 mL per well) and allowed to adhere for 12 h. The culture medium was then replaced with serum‐free medium, and the cells were subjected to serum starvation for 8–12 h. Subsequently, cells in the treatment groups were exposed to myristic acid at varying concentrations (5, 10, 50, and 100 µM) for 1 h. Following this, lipopolysaccharide (LPS, 1 µg/mL) was added to all groups except the control, and incubation continued for an additional 24 h. Finally, cells were lysed using TRIzol reagent, and total RNA was extracted for subsequent qPCR analysis [59].

### 5.13 Real‐Time Quantitative Reverse Transcription‐PCR (qRT‐PCR)

Total RNA was isolated from jejunal and colonic tissues using a commercial RNA extraction kit, followed by reverse transcription to synthesize cDNA. Gene expression levels of Rho, CDC42, BAIAP2, ZO‐1, Occludin, and MUC2 were quantified by RT‐qPCR. Primer sequences are listed in Table S3.

### 5.14 In Vitro Polysaccharide Growth Assays

*P. vulgatus* was grown anaerobically in BHI medium to the mid‐exponential phase (OD_600_ ≈ 0.8). Cultures were then transferred into pre‐reduced Minimal Medium (MM) supplemented with 0.5% (w/v) of the specified polysaccharides (Table S4). The bacterial suspension was diluted to an initial OD_600_ of 0.05, dispensed into 300 µL aliquots in multi‐well plates, and incubated anaerobically at 37°C. Growth was monitored using an Infinite F50 microplate reader by measuring OD_600_ every 0.5 h for 30 h. Polysaccharide background absorbance was corrected by subtracting the mean blank value from the raw OD_600_ readings, and the corrected values were used to plot growth curves. All cultures were performed in triplicate [84].

### 5.15 Whole‐Genome Sequencing and Analysis

Total genomic DNA was extracted from *P. vulgatus*, and sequencing libraries were prepared for Illumina and PacBio sequencing platforms and sequenced by Shanghai Majorbio Bio‐pharm Technology (Shanghai, China) at the 100 × coverage. Raw reads were quality trimmed with Fastp and assembled with Unicycler [85]. Genomic sequences of *P. vulgatus* were classified and annotated using the eggNOG and KEGG databases. Genome‐scale metabolic modeling was constructed based on MetaCyc pathways [86].

### 5.16 RT‐qPCR Quantification of Bacteria

To minimize potential PCR inhibition, the extracted DNA was diluted 1:1000 with sterile, nuclease‐free water prior to performing RT–qPCR. Primer sequences are provided in Table S3. Samples were allocated randomly across 96‐well plates, with two negative controls included per plate (extraction buffer and nuclease‐free water). qPCR reactions were set up in triplicate for each sample and carried out on a QuantStudio 12K Flex system using SsoAdvanced Universal SYBR Green Supermix, on a QuantStudio 12K Flex (Applied Biosystems) using SsoAdvanced Universal SYBR Green Supermix (Bio‐Rad, 1725270), with each sample run in triplicate. Data were analyzed in QuantStudio Design and Analysis Software v2.6.0, and Cq values were converted to gene copy numbers per mL or g using the standard curve.

### 5.17 Construction of *P. vulgatus* Knockout Strain

Mutant strains were generated using an inducible CRISPR‐Cas system following previously established protocols [32]. The *P. vulgatus* FTJS7K1 strain served as the parental background for creating GH16 and *fabB* knockout mutants. The genome editing workflow consisted of four main phases: plasmid construction, conjugation and selection, CRISPR‐Cas‐mediated gene deletion with verification, and plasmid curing. First, a *Bacteroides–E. coli* shuttle plasmid was generated using Gibson assembly. Single‐guide RNAs (sgRNAs) targeting GH16 and *fabB* were designed iusing Benchling (https://benchling.com/). Homology arms (∼1 kb) flanking the target regions were amplified with the primers listed in Table S3 and assembled with a linearized pBac001 backbone via seamless cloning. The resulting plasmids were introduced into E. coli S17‐1 λpir and verified by sequencing. Validated constructs were then transferred into *P. vulgatus* FTJS7K1 through conjugation. After anaerobic incubation, transformants were selected on GAM agar supplemented with appropriate antibiotics and cultured under the same conditions. Genome editing was induced by transferring cultures into selective medium containing anhydrotetracycline (aTc). Following induction, colonies were screened by PCR using primers listed in Table S3, and successful mutants were confirmed by sequencing. The edited strains were subsequently passaged in antibiotic‐free medium to eliminate the plasmid.

### 5.18 Quantification and Statistical Analysis

Data are presented as mean ± SEM. Statistical analyses were conducted using GraphPad Prism 9.0 and SPSS 27.0. Data distribution was assessed for normality prior to analysis. For normally distributed data, differences between two groups were evaluated using an unpaired t‐test, while comparisons among multiple groups were performed using one‐way ANOVA followed by appropriate post hoc tests based on variance homogeneity. For data that did not meet normality assumptions, nonparametric tests were applied, including the Mann–Whitney U test or Kruskal–Wallis test, as appropriate. A *p* value < 0.05 was considered statistically significant.

## Author Contributions

**Chuan Zhang**, **Miaoyu Li**, **Tiantian Li**, and **Fengwei Tian** designed the study protocol and supervised all parts of the project. **Xiaojie Zang** and **Haiyang Shi** conducted animal experiments. **Yaru Liu** performed the molecular biology experiments. **Chengcheng Zhang** did the bioinformatics analyses in close collaboration. **Fengwei Tian**, **Chengcheng Zhang**, **Jianxian Zhao**, **Qixiao Zhai**, and **Wei Chen** contributed to text revision and discussion.

## Funding

This work was financially supported by the National Key Research and Development Project (2022YFF1100203), the National Natural Science Foundation of China (32425044 and 32502150), the Jiangsu Funding Program for Excellent Postdoctoral Talent (2025ZB702), and the Collaborative Innovation Center of Food Safety and Quality Control in Jiangsu Province.

## Conflicts of Interest

The authors declare no conflicts of interest.

## Supporting information




**Supporting File 1**: advs78105‐sup‐0001‐SuppMat.xlsx.

**Supporting File 2**: advs78105‐sup‐0002‐SuppMat.docx.

## Data Availability

The data that support the findings of this study are openly available in National Genomics Data Center at https://ngdc.cncb.ac.cn, reference number PRJCA059621and PRJCA059621.
