# Supplementary material for: A Postbiotic‐Inducing Ganoderma applanatum Polysaccharide Repairs the Intestinal Barrier via a Phocaeicola vulgatus ‐ Tridecylic Acid Axis
Source: Adv Sci (Weinh). 2026 Oct 6:e78105. Online ahead of print. doi: 10.1002/advs.78105 (PMC13643241; doi:10.1002/advs.78105)
Supplement: Supplementary file 1 [file ADVS-9999-e78105-s002.docx]

**Fig. S1: Effects of GAPs on intestinal barrier-related enzyme activities and inflammatory markers.**

(A-C) The scatter plots show the concentrations of related enzymes—inducible nitric oxide synthase (iNOS), myeloperoxidase (MPO) and cyclooxygenase-2 (COX-2)—in the colonic tissues of colitis mice. (D-H) The immunological cytokines IL-1β, IL-6, IL-17, TNF-α, and IL-10 were measured in the colonic tissues of colitis mice. These data are presented for four fractions (GAP1, GAP2, GAP3, and GAP4) obtained by eluting water-extracted GAPs under a salt gradient, as well as the polysaccharide intragastric administration control group (Control) and the DSS-induced model group (Model) (n = 6 per group). One-way ANOVA with Tukey’s test was used for analysis of gene expression analysis. “ns” indicates *p* > 0.05; * indicates *p* < 0.05; ** indicates *p* < 0.01; *** indicates *p* < 0.001 and **** indicates *p* < 0.0001.

**Fig. S2: Monosaccharide composition of GAP1 and GAP2.**

(A) Ion chromatogram of monosaccharide standard (left) and GAP1 (right). (B) Ion chromatogram of monosaccharide standard (upper) and GAP2 (lower).

**Fig. S3: Isolation, purification and structural analysis of Polysaccharides GAP1 and GAP2 from *G. applanatum*.**

(A, B) The molecular weight and purity of the purified fractions GAP1 and GAP2 of GAPs were determined by high-performance gel-permeation chromatography (HPGPC), and the molecular weight distribution of GAPs was obtained. (C, D) Fourier transform infrared spectra of GAP1 and GAP2. (E-F) Two-dimensional NMR spectra of GAP1 and GAP2 were acquired using heteronuclear multiple bond correlation (HMBC).

**Fig. S4: Efficacy of GAP1 and GAP2 in the remission phase of DSS-induced colitis.**

(A) Changes in the content of tight junction proteins in the colon of DSS-treated mice after fecal microbiota transplantation (*n* = 6, per group). (B) Changes in the activity of inflammation-related enzymes in the colon of DSS-treated mice after fecal microbiota transplantation (*n* = 6, per group). (C) H&E and PAS staining of DSS-induced mice treated with microbiota-derived metabolites of GAP1 and GAP2 during the remission phase of colitis. One-way analysis of variance (ANOVA) combined with Tukey's test was used for gene expression analysis. “ns” indicates *p* > 0.05; * indicates *p* < 0.05; ** indicates *p* < 0.01; *** indicates *p* < 0.001 and **** indicates *p* < 0.0001.

**Fig. S5: Analysis of key effector bacteria and metabolites involved in GAP2-mediated repair of the intestinal barrier.**

(A) Comparison chart of differences in functional protein composition between DSS-induced mice (Model) and GAP2-gavaged mice (GAP2). (B, C) Metabolic pathway enrichment map and functional annotation map of fecal samples from DSS-induced colitis model mice (Model) and GAP2-gavaged mice (GAP2) (*n* = 6 per group).

**Fig. S6: Whole-genome analysis of *Phocaeicola vulgatus*.**

(A) Circular map of the whole genome of *Phocaeicola vulgatus* (FTJS7K1). (B) Circular display of the distribution of glycoside hydrolase (GH) families in *Phocaeicola vulgatus* (FTJS7K1). (C) Bar chart of COG functional classification of genes in *Phocaeicola vulgatus* (FTJS7K1). (D) Bar chart of KEGG functional classification of genes in *Phocaeicola vulgatus* (FTJS7K1).

**Fig. S7: Signal pathway diagram of fatty acid biosynthesis in *Phocaeicola vulgatus* (FTJS7K1)**

**Fig. S8: Differential gene analysis.** Using RNA-seq to explore the effect of GAP2 treatment on intestinal transcripts in DSS-induced colitis, showing a clustered heatmap of the top 30 enriched genes (*n* = 6 per group).

**Fig. S9: Analysis of the protective mechanism of GAP2 against DSS-induced colitis.**

(A, B) GO enrichment analysis and pathway enrichment analysis of downregulated genes in the feces of DSS-induced colitis mice administered with GAP2 by gavage (*n* = 6 per group). (C) GO enrichment analysis and pathway enrichment analysis of upregulated genes in the feces of DSS-induced colitis mice administered with GAP2 by gavage (*n* = 6 per group).
